# The impact of Lithium on thyroid function in Chinese psychiatric population

**DOI:** 10.1186/s13044-015-0026-2

**Published:** 2015-09-04

**Authors:** Kwan Yee Queenie Tsui

**Affiliations:** Department of Psychiatry, Pamela Youde Nethersole Eastern Hospital, 3 Lok Man Road, Chai Wan, Hong Kong SAR, China

**Keywords:** Lithium, Thyroid, Subclinical, Hypothyroidism, Psychiatric

## Abstract

**Background:**

Lithium was known to cause thyroid dysfunction and most commonly subclinical hypothyroidism (SCH). The aim of this study is to determine the prevalence of Lithium associated thyroid dysfunction and to identify risk factors associated with development of SCH in patients receiving Lithium.

**Methods:**

A retrospective cross-sectional study was conducted. Subjects who developed elated thyroid stimulating hormone (TSH) were compared with those who remained euthyroid with Lithium treatment. Logistic regression and survival analysis were applied to identify the significant factors associated with SCH.

**Results:**

The prevalence of Lithium associated with SCH was 31.7 %. The significant risk factors associated with increased risk of SCH included being female, higher serum Lithium level, concomitant use of Valproate Sodium and use of antidepressant. Use of depot injection was associated with decreased risk of SCH.

**Conclusions:**

Use of depot and avoidance of Valproate or antidepressant should be taken into account before starting patient on Lithium treatment. Thyroxine replacement should be considered when Lithium associated SCH was identified.

## Background

Lithium, discovered in 1817, was initially used for treating gout. Throughout years of research since its introduction, Lithium has now been well established as an effective agent for treatment in acute mania, prophylaxis of bipolar disorder and augmentation in refractory depression. Possible long term side effects included renal insufficiency, thyroid dysfunction, persistent tremor, and dermatologic effects of acne and alopecia [1]. Among the possible side effects of Lithium, thyroid dysfunction especially subclinical hypothyroidism (SCH) was a common yet often neglected one. Lithium was reported to concentrate in thyroid gland and inhibit thyroidal iodine uptake. It also inhibits iodotyrosine coupling, alters thyroglobulin structure and inhibits thyroid hormone secretion. Lithium inhibits synthesis and release of thyroid hormones by stabilizing effect on thyroid microtubules, decreasing adenylate cyclase responsiveness to TSH and suppressing cyclic adenosine mono phosphate (c-AMP) production. Lithium lowers de-iodination in the liver and decreases the clearance of free T4 [2].

According to ETA Guideline, SCH was characterized by TSH above the upper reference limit in combination with a normal free thyroxine (T4). Clinical hypothyroidism was characterized by TSH above the upper reference limit in combination with low free T4. Both situations were indicated by elated TSH.

The prevalence of clinical and subclinical hypothyroidism in the general population were 0.3 and 4.3 % respectively (NHANES III, 1988–1994). 36 % bipolar patients developed abnormal TSH with or without T4 abnormality while on Lithium [3]. The prevalence of clinical hypothyroidism while on Lithium was 10.4 % [4]. The prevalence of SCH while on Lithium was reported to be up to 20.3 % [5]. Bocchetta et al. noted only 1 case of hyperthyroidism among 150 patients on long term Lithium therapy during a 15 year follow up, suggesting Lithium induced hyperthyroidism was extremely rare [6]. Thus in this study, we would focus on mainly risk factors associated with development of elated TSH on Lithium.

## Methods

This study was a retrospective cross-sectional analysis of case notes of all Chinese psychiatric patients, who were receiving Lithium treatment and had serum thyroid function test (TFT) and Lithium level taken in the past one year on 1st March 2014. It was conducted in the Department of Psychiatry of Pamela Youde Nethersole Eastern Hospital (PYNEH) in Hong Kong. The medical records of potential study sample were retrieved and screened from the Medical Record Office (MRO) and electronic Clinical Management System (e-CMS) of Hospital Authority in Hong Kong. The reference range of TSH and free T4 were 0.35–3.80mIU/L and 9.5–18.1pmol/L respectively in PYNEH. Patients who were not from Chinese origin and had thyroid dysfunction before starting Lithium treatment as indicated by TFT or documented in medical notes were excluded. Among 262 patients eligible for the study, 109 (41.6 %) patients were male and 153 (58.4 %) were female. The study population comprised of relatively balanced gender ratio of 1 male to 1.4 female. The mean age of the study population was 48.57 years old with standard deviation of 12.267 years.

A collection of important factors were indentified as independent variables that might predispose to the development of SCH in the participants, including demographic factors, clinical characteristics and characteristics of medications prescribed. The number of months between the date of starting Lithium and development of elated TSH would be calculated as “duration of Lithium use”. If TSH remained normal throughout the study period, the number of months between the dates of starting Lithium till 1st March 2014 would then be calculated as “duration of Lithium use”.

### Data analysis

Comparison of demographic and clinical data between patients with normal and elated TSH while on Lithium treatment were done in univariate analysis. Variables showing statistically significant difference (*p* < 0.05) or marginally significant difference (0.05 ≤ *p* < 0.10) would be indentified and multivariate logistic regression analysis would then applied. Variables having *p* < 0.05 after multivariate logistic regression were considered as significant. Survival analysis was further conducted to indentify factors associated with development of elated TSH while on Lithium treatment. Kaplan-Meier survival curve would be plotted to give graphical representation of the survival function. Data was analyzed through IBM Statistical Product and Service Solution (SPSS) software version 22.

### Ethics approval

This study was approved by the Ethics Committee of Hong Kong East Cluster of Hospital Authority (HA) and the Chief of Service of the Department of Psychiatry of PYNEH.

## Results

**Fig. 1:**
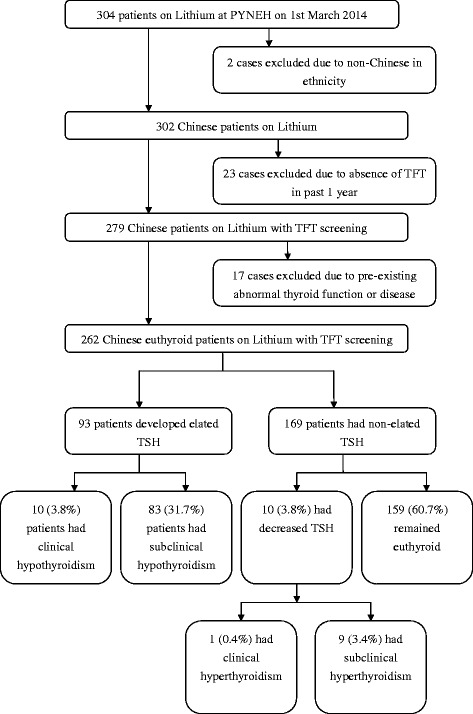
The forming of the study population after exclusion

Total 304 cases that were on Lithium treatment at PYNEH on 1st March 2014 were indentified through CDARS. Ethnicity, availability of thyroid and Lithium monitoring in the past one year and presence of thyroid illness before starting Lithium treatment were screened. Total 42 cases were excluded. The overall number of patients eligible for study was 262. Among the recruited patients, 93 (35.5 %) cases developed elated TSH, of whom 10 (3.8 %) cases had clinical hypothyroidism and 83 (31.6 %) cases had SCH. TSH of the remaining 169 (64.5 %) patients remained non-elated while on Lithium treatment. 10 (3.8 %) patients developed decreased TSH, of whom 1 (0.4 %) case developed clinical hyperthyroidism and 9 (3.4 %) cases developed subclinical hyperthyroidism. 159 (60.7 %) patients remained euthyroid throughout the study. Figure 1 showed the forming of the study population.

Univariate analysis was preformed between patients with elated and non-elated TSH for demographic factors, clinical characteristics and characteristics of medications prescribed. Gender showed significant value of *p* = 0.005. Use of Valproate Sodium (*p* < 0.001) and use of depot injection (*p* = 0.008) were statistically significant and types of Lithium taking (*p* = 0.073) was marginally significant. Serum Lithium level (*p* = 0.001) was also statistically significant. The univariate test results were summarized in Table 1.

**Table 1:** Univariate tests for factors between patient with non-elated and elated TSH

Factors	Non-elated TSH (*N* = 169)	Elated TSH (*N* = 93)	*p*
Male	81 (47.9 %)	28 (30.1 %)	0.005*^a^
Female	88 (52.1 %)	65 (69.9 %)	
Primary Psychiatric Diagnosis			0.573^b^
Schizophrenia/Schizoaffective	51 (30.1 %)	33 (35.5 %)	
Disorder/Psychosis			
Bipolar Affective Disorder/Mania	86 (50.9 %)	46 (49.5 %)	
Depression/Anxiety	26 (15.4 %)	11 (11.8 %)	
Mental Retardation	3 (1.8 %)	3 (3.2 %)	
Others	3 (1.8 %)	0 (0.0 %)	
Secondary Psychiatric Diagnosis			0.309^b^
Personality Disorder	1 (0.6 %)	1 (1.1 %)	
Mental Retardation	6 (3.5 %)	1 (1.1 %)	
Substance Abuse	5 (3.0 %)	1 (1.1 %)	
Autistic Spectrum Disorder	2 (1.2 %)	1 (1.1 %)	
Others	0 (0.0 %)	2 (2.1 %)	
Nil	155 (91.7 %)	87 (93.5 %)	
Physical Comorbidities			
Hypertension			0.505^a^
Yes	25 (14.8 %)	11 (11.8 %)	
No	144 (85.2 %)	82 (88.2 %)	
Diabetes			0.911^a^
Yes	21 (12.4 %)	12 (13 %)	
No	148 (87.6 %)	81 (87 %)	
Hyperlipidaemia			0.410^a^
Yes	18 (10.7 %)	7 (7.5 %)	
No	151 (89.3 %)	86 (92.5 %)	
Cardiovascular disorders			0.617^b^
Yes	2 (1.2 %)	2 (2.2 %)	
No	167 (98.8 %)	91 (97.8 %)	
Types of Lithium taking			0.073**^a^
Lithium CR	141 (83.4 %)	69 (74.2 %)	
Lithium IR	28 (16.6 %)	24 (25.8 %)	
Frequency of Lithium per day			0.311^b^
Once	147 (87.0 %)	75 (80.6 %)	
Twice	20 (11.8 %)	17 (18.3 %)	
Three times	2 (1.2 %)	1 (1.1 %)	
Use of depot injection			0.008*^b^
Yes	22 (13.0 %)	3 (3.2 %)	
No	147 (87.0 %)	90 (96.8 %)	
Use of oral antipsychotics			0.421^a^
Yes	129 (76.3 %)	75 (80.6 %)	
No	40 (23.7 %)	18 (19.4 %)	
Use of antidepressant			0.746^a^
Yes	62 (36.7 %)	36 (38.7 %)	
No	107 (63.3 %)	57 (61.3 %)	
Use of Benzodiazepine			0.742^a^
Yes	80 (47.3 %)	46 (49.5 %)	
No	89 (52.7 %)	47 (50.5 %)	
Use of Carbamazepine			0.669^b^
Yes	3 (1.8 %)	3 (3.2 %)	
No	166 (98.2 %)	90 (96.8 %)	
Use of Valproate Sodium			< 0.001*^a^
Yes	18 (10.7 %)	31 (33.3 %)	
No	151 (89.3 %)	62 (66.7 %)	
Use of other mood stabilizers			1.000^a^
Yes	5 (3.0 %)	2 (2.2 %)	
No	164 (97.0 %)	91 (97.8 %)	
Use of non-psychiatric medications			0.786^a^
Yes	77 (45.6 %)	44 (47.3 %)	
No	92 (54.4 %)	49 (52.7 %)	
Age, mean (SD)	48.47 (12.188)	48.76 (12.473)	0.852^c^
Serum Lithium Level (μmol /L), mean (SD)	541.30 (217.701)	639.14 (212.984)	0.001*^c^
Dosage of Lithium (mg), median (IQR)	800 (675–1000)	800 (600–800)	0.364^d^

*Note*: *significant *p* < 0.05, **marginally significant 0.05 ≤ *p* < 0.10, ^a^Chi-squared test, ^b^Fisher’s exact test, ^c^Independent-sample-*t*-test, ^d^Mann-Whitney *U* Test, *SD* Standard Deviation, *IQR* Interquartile Range

Multivariate logistic regression was then applied. Gender (*p* = 0.001) was significantly associated with development of elated TSH. Male gender was associated with lower odds of development of elated TSH, in which the odds of developing elated TSH would be 37 % of that in female. Serum Lithium level (*p* = 0.007) was also found to be associated with higher odds of development of elated TSH. One unit (1 μmol /L = 0.001 mEq/L) increase was associated with 0.2 % increase in the odds of developing elated TSH (OR = 1.002). Moreover, co-administration of depot injection with Lithium (*p* = 0.013) was shown to significantly lower risk of developing elated TSH. The odds of developing elated TSH for those on depot injection was 18.9 % of those not on depot injection (OR = 0.189). When Valproate Sodium was prescribed with Lithium (*p* < 0.001), there would be an increased risk of developing elated TSH. Patients on Valproate would have 4.522 times greater risk in having elated TSH than those not on Valproate (OR = 4.522). The multivariate logistic regression results were shown in Table 2.

**Table 2:** Significant factors identified in multivariate analysis

Multivariate Logistic Regression		
Factors	OR (95 % CI)	*p*
Gender (Male)	0.370 (0.202–0.679)	0.001
Serum Lithium Level	1.002 (1.001–1.003)	0.007
Use of depot injection	0.189 (0.051–0.704)	0.013
Use of Valproate Sodium	4.522 (2.179–9.381)	< 0.001
Survival Analysis		
Multivariate Cox Regression		
Factors	HR (95 % CI)	*p*
Gender	0.541 (0.344–0.851)	0.008
Serum Lithium Level	1.001 (1.0–1.002)	0.015
Use of depot injection	0.308 (0.097–0.978)	0.046
Use of antidepressant	1.576 (1.021–2.435)	0.040
Use of Valproate Sodium	2.056 (1.311–3.224)	0.002

*Note:* Significant *p* < 0.05, *OR* Odds Ratio, *HR* Hazard Ratio, *CI* Confidence Interval

**Fig. 2:**
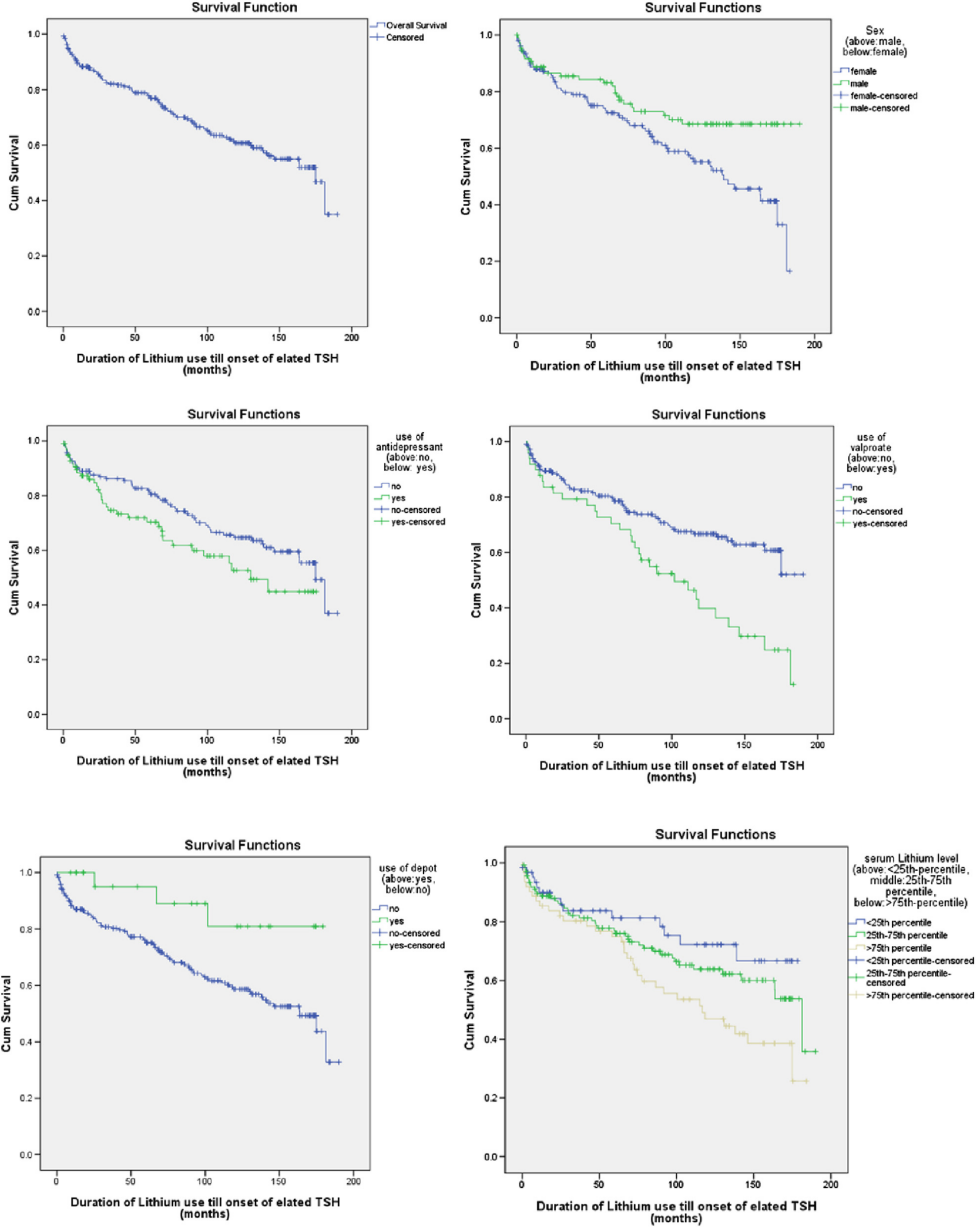
Kaplan-Meier survival curves showing the significant results in Survival Analysis

After logistic regression analysis, survival analysis was further conducted to explore factors which correlated to the time to development of elated TSH. Univariate cox regression was applied to all the variables with results shown in Table 3. Multivariate cox regression was then applied to the factors having *p* < 0.10 in univariate cox regression. The multivariate cox regression identified the same four significant factors as the previous multivariate logistic regression analysis except one extra factor ‘concomitant use of antidepressant’ was identified as significant in survival analysis. The results of multivariate cox regression were presented as follows. Firstly, gender (*p* = 0.008) was statistically significant, and the hazard of male patients in developing elated TSH was 54.1 % of that in female patients (HR = 0.541). Secondly, serum Lithium level (*p* = 0.015) was associated with higher hazard in developing elated TSH, and one unit (1 μmol/L = 0.001 mEq/L) increase was associated with 0.1 % raise in hazard of developing elated TSH (HR = 1.001). Thirdly, depot injection (*p* = 0.046) was shown to significantly lower the risk in developing elated TSH while on Lithium, and the hazard of patients using depot was 30.8 % of those not on depot injection (HR = 0.308). Fourthly, use of Valproate Sodium was significant (*p* = 0.002), and the hazard of patients on Valproate Sodium was 2.056 times greater in developing elated TSH than those not taking (HR = 2.056). Lastly, as described above, concomitant use of antidepressant with Lithium (*p* = 0.04) was noted to be a significant factor, and the hazard of patients using antidepressant in developing elated TSH was 1.576 times greater than those not on antidepressant treatment (HR = 1.576). The multivariate cox regression results were also shown in Table 2. In addition, Kaplan-Meier survival curves were plotted for the significant results identified in survival analysis as shown in Fig. 2.

**Table 3:** Univariate cox regression for survival analysis

Demographic factors	*p*
Gender	0.011*
Age	0.726
Clinical Characteristics	
Primary Psychiatric Diagnosis	0.992
Secondary Psychiatric Diagnosis	0.296
Hypertension	0.136
Diabetes	0.405
Hyperlipidaemia	0.243
Cardiovascular disorders	0.833
Characteristics of medications prescribed	
Types of Lithium taking	0.109
Frequency of Lithium per day	0.298
Serum Lithium Level	0.028*
Dosage of Lithium	0.762
Use of depot injection	0.035*
Use of oral antipsychotics	0.908
Use of antidepressant	0.067**
Use of Benzodiazepine	0.588
Use of Carbamazepine	0.221
Use of Valproate Sodium	0.001*
Use of other mood stabilizers	0.577
Use of non-psychiatric medications	0.449

*Note*: *Significant *p* < 0.05, **Marginally Significant 0.05 ≤ *p* < 0.10

## Discussion

In this study, 35.5 % patients developed elated TSH while on Lithium treatment and it was close to the rate (36 %) done in Pittsburgh study comprising of 143 patients [3]. Lithium induced clinical hyperthyroidism was shown to be a rare event as only 1 out of 262 patients developed clinical hyperthyroidism in this study.

It was found that the risk of developing SCH when on Lithium was significantly higher in women, which was also reported in other studies [4, 7]. Such difference was likely due to the exacerbating effect of estradiol in women and protective effect of testosterone in men. It has been demonstrated that estradiol would decrease iodide uptake in thyroid follicular cells [8]. Testosterone had also been shown to decrease thyroid enlargement and prevent the fall in free T4 levels in rats [9].

It was demonstrated in this study that there was a positive correlation between serum Lithium level and risk of developing elated TSH. A few studies had also reported on such finding [10, 11].

Antipsychotics were well known for their central activity on tuberoinfundibular dopaminergic pathway. The antagonism of dopamine 2 receptor could increase Thyroid Releasing Hormone stimulated TSH levels [12]. Use of depot injection had been shown to decrease risk of developing SCH. This was probably related to more stable plasma antipsychotics concentration with less fluctuation when using depot rather than oral administration [13].

When Valproate Sodium was taken concomitantly with Lithium, the risk of developing SCH would become greater. This showed that Lithium and Valproate Sodium had synergistic effect in inducing SCH. Valproate alone had been proven to induce SCH by several studies [14]. Valproate had been reported to increase serum Gamma-aminobutyric Acid (GABA) level [15]. GABA was also shown to inhibit TSH-stimulated thyroid hormone release from the thyroid gland [16]. Another mechanism proposed was Valproate induced SCH through blockage of N-methyl-D-aspartate (NMDA) synaptic transmission [17], while NMDA had been shown to increase serum thyroid hormone level [18]. Hence, both mechanisms would stimulate TSH release from the pituitary gland.

At last, use of antidepressant with Lithium was shown to increase risk of developing SCH. Such increased in risk was consistent with previous study results in which patients developed increased TSH while on Sertraline and Desipramine [19, 20]. Though the mechanism behind remained unclear. The possible explanation would be serotonin increased by antidepressant when administered in a chronic fashion induced lower plasma levels of thyroid hormones [21]. Thus, Lithium and antidepressant had synergistic effect in inducing SCH and such effect was only revealed by survival analysis when survival time was taken into account.

For patients who were at increased risk of developing thyroid dysfunction including being female, with personal or family history of thyroid disorders and with positive autoimmune markers or abnormal TFT in the initial blood taking prior to initiating Lithium, special caution in prescription regimen to avoid further exacerbation or development of thyroid dysfunction should be taken. According to this study, depot injection could be put at a higher priority than oral administration when antipsychotics were indicated. Simplification of regimen should be adopted, since co-administration of Valproate Sodium or antidepressant had been shown to increase risk of developing SCH. When antidepressant was indicated, it should be prescribed for short term use if possible since chronic administration would have synergistic effect with Lithium in inducing SCH. Over-treatment should be avoided and lowering Lithium dosage after acute episode should be considered, since there was an increased in risk of developing SCH for every unit increase in serum Lithium level.

Discrepancy existed between different guidelines in reference to ongoing TFT monitoring in patients taking Lithium. National Institute for Health and Care Excellence (NICE), British National Formulary (BNF) and South London & Maudsley (SLAM) recommended TFT to be taken every 6 months while Best Practice review series and Drugs & Therapeutic Bulletin (DTB) recommended TFT to be taken every 12 months. Lithium had been shown to increase antibody titers, especially, when these antibodies had been present at the start of treatment [22]. However, among all the above guidelines, antiperoxidase and antithyroglobulin measurements had not been included in the initial blood tests recommended prior to start of Lithium. Also compliance to recommended guidelines were still difficult when requirements were too stringent, such as only 35 % of patients were compliant to the recommended guideline of three-monthly plasma Lithium level taking in 2013 NICE audit. Thus balance between theoretical recommendations and practical clinical scenarios remained for further discussion.

Patient’s mental state should be closely monitored when put on Levothyroxine treatment since it had been reported that some patients would experience mania while on replacement therapy [23]. Levothyroxine treatment should be started prior to antidepressant trial for bipolar patients with depressive symptoms associated with SCH since thyroxine replacement might lead to improvement in mood.

To our knowledge, this is the first study to have extensively investigated on risk factors and predictors on Lithium associated SCH in Chinese patients. Apart from gender and age which had been widely investigated in previous studies, other risk factors including concomitant use of various psychiatric medications, comorbidities and serum Lithium level had been comprehensively investigated in this study by both logistic regression and survival analysis.

For the study results, consistent risk factors were found using both logistic regression and survival analysis meaning that these risk factors remained stable and significant despite employment of different statistical analyses. These risk factors not only showed statistical significance, but also gave clinical significance in further revision of current clinical guidelines and recommended treatment in terms of Lithium associated SCH.

There were several limitations in this study. The retrospective study design confined the collection of data to be only obtained from the case records. Other factors including infection, stress and smoking could also induce thyroid dysfunction but such information had not been recorded properly and consistently in case notes. Another limitation was this study was unable to develop causality between exposure of the risk factors in development of Lithium associated SCH. Besides, the severity of psychiatric diseases, which might had an effect on the results apart from the pharmacological action of drugs, had not been investigated in this study. The number of individuals having various severity levels of different psychiatric diagnoses would be too small and therefore they had been grouped together according to their mental disorders for statistical analysis.

Further research is required to establish and investigate on the effectiveness of depot in reducing development of SCH on Lithium when compared with use of oral antipsychotics. The effect of chronic administration of antidepressant and Lithium should also be further explored in development of SCH. Few studies had investigated on relationship with serum Lithium level and risk of SCH and further research might be required. With research in more comprehensive and deeper perspective, it was hoped that further improvement in current Lithium guidelines could be reached.

## Conclusions

Despite various psychiatric medications had been discovered and manufactured throughout the century, Lithium remained the only drug with an established anti-suicidal efficacy for affective disorders. It was still widely advocated in patients with bipolar affective disorder and refractory depression. Thyroid dysfunction complicated by Lithium treatment had been investigated, but SCH with related risk factors was often neglected. Use of depot and avoidance of Valproate or antidepressant should be taken into account for patients receiving Lithium treatment. Serum Lithium level and thyroid function should be monitored regularly, and thyroxine replacement should be considered when Lithium associated SCH was identified. With increasing evidence in harm and risk of untreated SCH, further research and revision of current Lithium guidelines would be required.
